# Population transcriptomics uncovers the regulation of gene expression variation in adaptation to changing environment

**DOI:** 10.1038/srep25536

**Published:** 2016-05-06

**Authors:** Qin Xu, Caiyun Zhu, Yangyang Fan, Zhihong Song, Shilai Xing, Wei Liu, Juan Yan, Tao Sang

**Affiliations:** 1 Key Laboratory of Plant Resources and Beijing Botanical Garden, Institute of Botany, Chinese Academy of Sciences, Beijing 100093, China; 2 State Key Laboratory of Systematic and Evolutionary Botany, Institute of Botany, Chinese Academy of Sciences, Beijing 100093, China; 3 University of Chinese Academy of Sciences, Beijing 100049, China; 4 Key Laboratory of Plant Germplasm Enhancement and Speciality Agriculture, Wuhan Botanical Garden, Chinese Academy of Sciences, Wuhan, Hubei 430074, China

## Abstract

Expression variation plays an important role in plant adaptation, but little is known about the factors impacting the expression variation when population adapts to changing environment. We used RNA-seq data from 80 individuals in 14 *Miscanthus lutarioriparius* populations, which were transplanted into a harsh environment from native habitat, to investigate the expression level, expression diversity and genetic diversity for genes expressed in both environments. The expression level of genes with lower expression level or without SNP tended to be more changeable in new environment, which suggested highly expressed genes experienced stronger purifying selection than those at lower level. Low proportion of genes with population effect confirmed the weak population structure and frequent gene flow in these populations. Meanwhile, the number of genes with environment effect was the most frequent compared with that with population effect. Our results showed that environment and genetic diversity were the main factors determining gene expression variation in population. This study could facilitate understanding the mechanisms of global gene expression variation when plant population adapts to changing environment.

The molecular and physiological phenotypes of plant, such as protein levels or trait phenotypes can be coordinated by genetic and environmental factors via *cis-* or *trans-* expression regulation and environmentally induced epigenetic regulation^1^,^2^,^3^,^4^,^5^,^6^. Therefore, gene expression level is considered an intermediate phenotype from which conclusions about the emergence of high level traits can be drawn^7^,^8^. Generally, abrupt environmental changes can induce the change of gene expression programs, which can help adjust the regulation of plant growth and metabolism to the new environment in return and increase population-level phenotypic variation^9^,^10^,^11^,^12^,^13^. The increase of phenotypic variation is capable of producing alternative phenotypes and improving the potential of genetic evolution due to artificial and natural selection, and thus enables the evolution of heritable adaptive traits^14^,^15^. Hence, modulation of gene expression level has a central role in plant architecture and adaptation to changing environment. Exploring the characterization of population gene expression variation in changing environment is very important for understanding the roles and mechanisms of gene expression in plant adaptation to new environment.

Expression variation among populations is found to be pervasive in many plants such as maize, tomato and grapevine^9^,^16^,^17^,^18^,^19^,^20^,^21^. Those studies address the variation in gene expression within and between natural populations for studying genome evolution. Substantial expression variations, even among isogenic individuals or cells in seriously controlled common environment, can still be observed in response to different environmental conditions^9^,^22^. In general, gene expression level can be considered as a quantitative trait since it differs between individuals with respect to genetic factors, based on which many studies had mapped the genetic variation determining expression^1^,^23^,^24^. However, environment has been shown to have a stronger effect on gene expression than genetic variation^17^,^25^. The powerful functionality of environment on gene expression may be due to cryptic genetic variation, which has no discernable effect under common ecological environment and can be amplified in new stressful environment, facilitating evolutionary adaptation if this variation happens to be pre-adapted to a new environment and revealing advantageous^26^,^27^,^28^,^29^,^30^,^31^. Epigenetic modifications are another source of expression variation^32^. The establishment and maintenance of epigenetic modifications can contribute to the variability of phenotypes by regulating gene expression in response to the stress stimulation^33^.

For the past 40 years, it has gained wide acceptance that the genetic variation rate is determined mainly by the importance of protein function^34^,^35^. It has also been reported that expression abundance could negatively induce about 20–40% of variation in protein evolutionary rates^36^. In addition, genetic variation rates seem to correlate strongly with measures related to expression level and expression variation^37^,^38^. Many studies find that highly expressed genes have been under strong selective constraint for translation robustness^39^, and broadly expressed genes have been constrained by the need of function in several biochemical environments^40^.

Although these hypotheses are proposed, the genome-wide gene expression variation pattern has not been well characterized when population adapts to new environment. With the fast development of next generation sequencing platforms, high throughput transcriptome sequencing makes exploring global gene expression variation possible. In our previous experiment, we collected 14 populations of *Miscanthus lutarioriparius*, the candidate wild progenitor of second-generation energy crops, across its natural distribution and planted them in two experimental fields, one near its native habitat in Jiangxia of Hubei Province (JH) and the other at the domestication site Qingyang of Gansu Province (QG) with much colder and drier climates and poorer soil conditions. As a C4 perennial grass capable of producing high biomass on marginal land, *Miscanthus* has emerged to be a promising candidate of dedicated energy crops^41^,^42^,^43^,^44^,^45^,^46^. The following question is how to achieve fast adaptation to marginal and harsh land for *Miscanthus.* Field experiment showed that *M. lutarioriparius* was able to establish and to produce higher biomass in stressful environment than in native habitat^45^. To characterize the transcriptomics of *M. lutarioriparius*, a total of 40 individuals originated from the same natural populations and at the same growth stage were sampled from each field site for RNA-seq respectively. The comparison of population transcriptomes had demonstrated that a number of gene expression levels changed across environments^47^,^48^. Genetic diversity significantly decreased and the expression diversity significantly increased when population was transplanted from the native habitat to the unfavorable and harsh environment.

However, these above studies did not focus on characterizing the patterns and factors impacting gene expression variation. Moreover, previous studies of gene expression variation usually adopted the samples under strictly controlled environment, which suppressed the variability in population level^49^. Here we used population transcriptome of two natural field conditions imbuing various levels of variation to uncover the global gene expression pattern and expression variation in the process of adaptation. We aimed to carry out a thorough and comprehensive analysis to elucidate the expression variation pattern. In detail, we explored the relationship among the gene expression level (*E*_p_, see method), expression variation (*E*_p_ ratio), expression diversity (*E*_d_), and genetic diversity (π ) based on population transcriptomes. We tried to answer the question that what factors determine or correlate with the gene expression variation when plant population adapts to a changing environment.

## Results

### Expression of lower expressed genes tended to be more changeable in new environment

**Figure 1:**
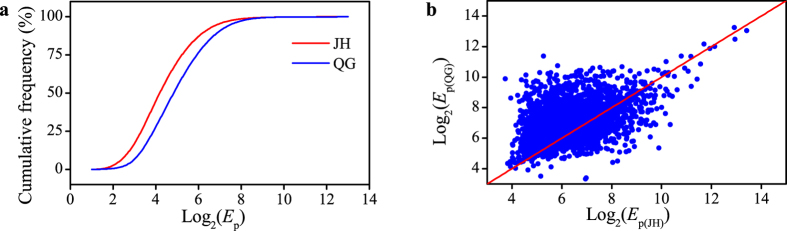
The comparison of gene expression abundance in population between JH and QG. (**a**) The cumulative frequency of gene expression abundance in JH and QG. *E*_p_s were transformed to the logarithm to the base 2. (**b**) The correlation of *E*_p_s for genes between JH and QG.

Expression level (*E*_p_, see method) and expression diversity (*E*_d_, see method) in the population were analyzed based on 40 *M. lutarioriparius* individuals from each environment. There were 15367 genes expressed in at least half of the individuals in each environment, and 11400 of them were expressed in all the individuals at both sites. The distribution of *E*_p_ for all genes within each environment was represented in Fig. 1. It was shown that *E*_p_s in transplanted or target domestic site QG were higher than those in near native habitat JH (Fig. 1a), and they were significantly related with each other between the two distinct environments (r =  0.98, *P* <  0.01) (Fig. 1b). To study the expression variation between two sites, we calculated *E*_p_ ratio for each gene (*E*_p(QG)_/*E*_p(JH)_). There were 58.3% (8961 genes) of genes having conserved *E*_p_ (*E*_p_ ratio ranged from 0.5 to 2) in new environment. Enriched Pfam group of these genes mainly included Ubiquitin-conjugating enzyme, FKBP-type peptidyl-prolyl cis-trans isomerase, Zinc finger, C3HC4 type (RING finger), RNA recognition motif, (Aka RRM, RBD, or RNP domain), Ubiquitin family, and Thioredoxin, all of which had more than 75% members having conserved *E*_p_ value (Supplementary Table S1). Using GO functional classification, we found these genes were mainly ATP hydrolysis coupled proton transport, acid-amino acid ligase activity, unfolded protein binding, transcription initiation, small GTPase mediated signal transduction, transcription initiation, protein folding, protein modification process, metalloendopeptidase activity, cell redox homeostasis and cellular protein metabolic process.

**Figure 2:**
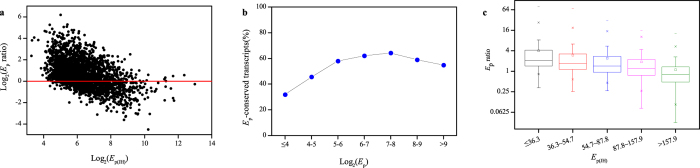
The comparison of gene expression abundance in population and expression variation between two distinct field sites. (**a**) The log_2_ (*E*_p_ ratio) was plotted log_2_*E*_p_ value for gene in JH, and *E*_p_ ratio for each genes was calculated using *E*_p(QG)_/*E*_p(JH)_. (**b**) The proportion of genes with conserved *E*_p_ between the two distinct field sites. The *E*_p_ ratio ranging from 0.5 to 2 was considered as conserved *E*_p._ (**c**) The relationship between *E*_p_s in JH and *E*_p_ ratio. The data was divided equally into five groups according to *E*_p_s in JH.

To analyze the relationship between expression variation and expression level, *E*_p_ ratio was plotted against *E*_p_s in near native habitat site JH (Fig. 2a). It was found that *E*_p_ ratio tended to decrease with the increase of *E*_p_s in JH. However, the percentage of genes with conserved *E*_p_ did not persistently increased along with the increase of *E*_p_, and reached the highest value (65%) when log_2_ (*E*_p_) was at 8. In order to obtain a significance test for the relationship between *E*_p_s and *E*_p_ ratio, all genes were divided into 5 groups, each of which had the same number of genes, according to their *E*_p_s value in JH (Fig. 2c). It was shown that *E*_p_ ratio significantly decreased with the increase of *E*_p_ between any two groups (Wilcoxon-test, *P* <  0.001). *E*_p_ ratios for the group with lower *E*_p_ value was significantly larger than that with higher *E*_p_ value, which suggested that highly expressed genes were relatively insensitive to environmental change.

### Expression level of genes with SNP was lower than that without SNP

**Figure 3:**
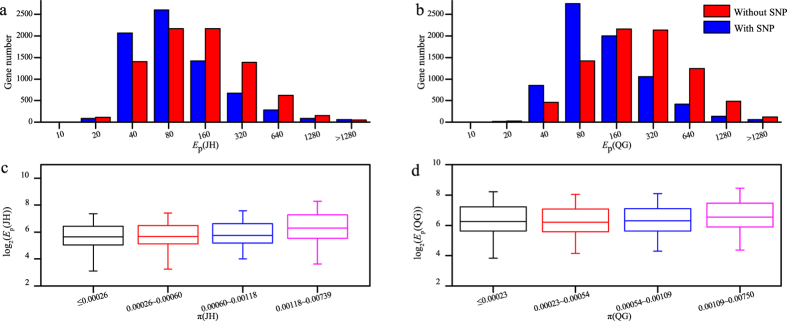
The relationship between gene genetic diversity and expression abundance in population. In each environment site, the genes were divided into two groups based on presence or absence of SNP. The distribution of *E*_p_s for each group was shown in (**a**) JH and (**b**) QG. The genes harboring SNP in each environment were divided into four groups according to their genetic diversity π . The distribution of *E*_p_ for genes in each divided group was shown in (**c**) JH and (**d**) QG.

We analyzed the relationship between expression level (*E*_p_) and genetic diversity (π ). Of the 15367 genes, only 7226 and 7248 genes had genetic variation (SNP) in near native habitat JH and transplanted site QG respectively. The genetic diversity (π ) ranged from 5.96E-06 to 7.39E-03 in JH and from 5.56E-06 to 7.50E-03 in QG, with an average value of 8.57E-04 and 7.98E-04, respectively. Based on the presence or absence of SNP, genes were grouped into two categories, one representing genes with SNP and the other representing genes without SNP. First, we tested whether the *E*_p_s were significantly different between gene groups with and without SNP. The distribution of *E*_p_s showed that the group without SNP had a greater number of genes than that with SNP when *E*_p_s were at a given level of 160 or greater both in JH and in QG, suggesting that *E*_p_s for genes without SNP tended to be larger than those with SNP (Fig. 3a,b). Further test of significance showed a significant difference of *E*_p_s between the two gene groups (Wilcoxon test, *P* <  0.001). These indicated that the genes with SNP usually expressed at a lower level.

Then we further tested the relationship between *E*_p_s and π . The *E*_p_s was plotted against π using the gene sets with SNP. Although it seemed low correlation between them, we further statistically tested the relationship by dividing the gene sets into four groups, each of which included the same number of genes. There were no significant difference in *E*_p_s between the first 3 groups, but the *E*_p_s of last group were significantly higher with the increase of π both in JH and in QG (Fig. 3c,d, Wilcoxon test, *P* <  0.01). In addition, since a longer sequence may have higher possibility of presence of SNP, we tested whether SNP was correlated with sequence length. It was shown that the distributions of gene length for the genes with or without SNP were almost consistent, which suggested that the presence or absence of SNP had no correlation with gene length. Thus, the contribution of genetic variation to expression level in population was mainly due to the presence or absence of SNP rather than the level of genetic diversity.

### Genes with lower genetic diversity tended to have higher expression diversity

**Figure 4:**
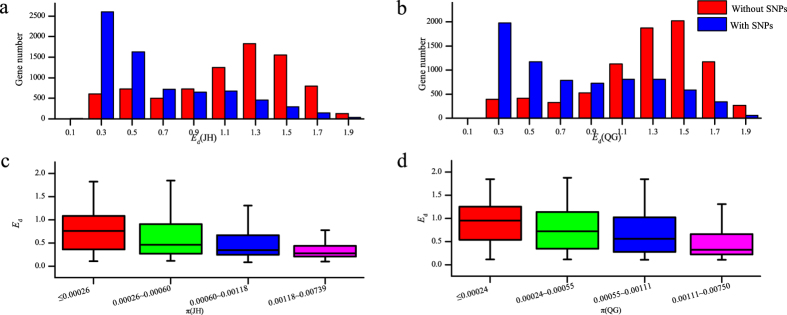
The relationship between gene genetic diversity and expression diversity in population. The genes were divided into two groups based on presence or absence of SNP. The distribution of gene expression diversity for each group was shown in (**a**) JH and (**b**) QG. The genes harboring SNPs in each environment were divided into four groups according to the π value, and the distribution of the expression diversity for genes in each group was shown in (**c**) JH and (**d**) QG.

To address the relationship between expression diversity (*E*_d_) and genetic diversity (π ), first we tested whether expression diversity was correlated with presence or absence of SNP. The group with SNP had a greater number of genes at a low level of *E*_d_ and a fewer number of genes at a high level of *E*_d_ both in JH and in QG when compared with the group without SNP (Fig. 4a,b). This suggested that the expression diversity of genes with SNP was always smaller than those without SNP. Using significance test, we found that it was significantly different (Wilcoxon test, *P* <  0.001). This result suggested that the genes without SNP always had a broader range of expression level in population than those with SNP.

To further test the relationship between expression diversity and genetic diversity, we divided genes with SNPs into 4 groups according to the range of π value, each of which had the same number of genes. It was shown that *E*_d_s decreased along with the increase of π both in JH and QG (Fig. 4c,d). Using significance test method, we found it was significantly different between any two groups (Wilcoxon test, *P* <  0.001). Together, our results showed that expression diversity was negatively correlated with genetic diversity.

### Expression level of gene with lower genetic diversity tended to be changed more easily in new environment

**Figure 5:**
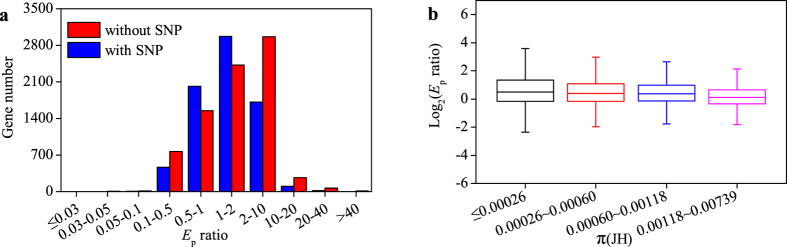
The relationship between genetic diversity and *E*_p_ ratio. (**a**) The genes were divided into two groups based on presence or absence of SNP. The number of gene in a specific range of *E*_p_ ratio was shown. (**b**) The genes harboring SNPs in each environment were divided into four groups according to π value in JH. The distribution of *E*_p_ ratio value was shown.

In order to examine the relationship between expression variation and genetic diversity, first we compared the *E*_p_ ratio between genes with and without SNP. The distribution of *E*_p_ ratio showed that the group without SNP had a greater number of nonconserved-*E*_p_ genes (*E*_p_ ratio <  0.5 or *E*_p_ ratio >  2) and a fewer number of conserved-*E*_p_ genes (0.5 <  *E*_p_ ratio <  2) when compared with the group with SNP (Fig. 5a), and this difference was significant (Wilcoxon test, *P* <  0.01). This suggested that the expression level of the genes without SNP were more easily to be changed in new environment compared with those with SNP.

Secondly we analyzed the relationship between expression variation and genetic variation by plotting *E*_p_ ratio against π for the genes set with SNP (Fig. 5b). All the genes harboring SNP were divided into 4 groups, each of which had the same number of genes. It was found that the *E*_p_ ratio decreased along with the increase of π . Using significance test, we found that it was significantly different between any two groups except for the comparison of the middle two (Wilcoxon-test, *P* <  0.001). Together, these results showed that the expression variation tended to decrease with the increase of genetic diversity, which suggested that genes with lower genetic diversity were more sensitive to environmental change at expression level.

### Environment was the leading contributor to gene expression variation compared with population and haplotype

**Figure 6:**
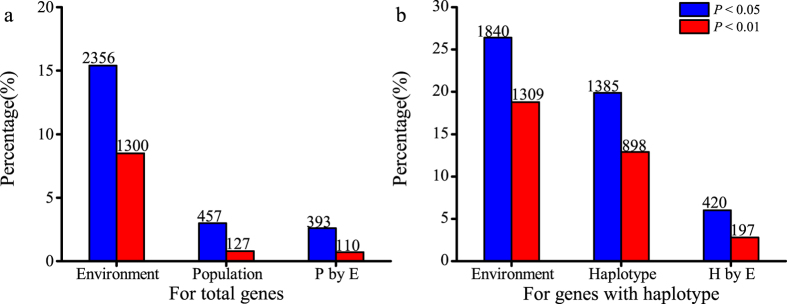
Comparison of genes with environment effect, population effect, haplotype effect and their interaction effect. (**a**) All transcripts were used for analysis of the genes with environment effect, population effect, and their interaction effect. P by E indicates the genes with population by environment effect; (**b**) The phased gene set were used for analysis of the genes with environment effect, haplotype effect, and their interaction effect. H by E indicates the genes with haplotype by environment effect. Significance test was set at the level of *P* <  0.05 (blue) and *P* <  0.01 (red).

We performed analysis of variation (ANOVA) to evaluate the factors impacting gene expression variation using the groups of two environments and 14 populations. Out of 15367 genes, we found that 2365 (15.4%), 457 (3.0%) and 393 (2.6%) genes had environmental effect, habitat population effect, and population by environment effect on the expression variation, respectively (*P* <  0.05) (Fig. 6a). Enriched Pfam group of these genes was also shown in Supplementary Table S2. This suggested that environment played a much broader role in gene expression variation than population effect.

In addition, we phased the haplotypes based on the population transcriptome data to analyze the haplotype effect. Totally 6964 genes were phased for ANOVA of environment and haplotype effects on gene expression variation. It was found that 1840 (26.4%), 1385 (19.9%) and 420 (6.0%) genes had environment, haplotype and haplotype by environment effect, respectively (Fig. 5b, Supplementary Table S3, *P* <  0.05), which suggested that environment played a more important role in gene expression variation than haplotype. Together, environment played the leading role in gene expression variation compared with population and haplotype.

To compare the relative effect of population and haplotype on expression variation, we compared the percentage of genes with population effect and haplotype effect. 3.0% (457 genes) and 19.8% (1385 genes) out of their gene sets were identified to have population effect and haplotype effect, respectively (Fig. 5, Supplementary Table S3, *P* <  0.05), which suggested that genotype effect (haplotype) was more than population effect on gene expression variation.

Since environment and haplotype were the two leading factors impacting gene expression, we classified genes into 8 groups according to whether genes had one or more of the environment, haplotype or their interaction effects (Supplementary Fig. S1, Supplementary Table S3). We found that genes with different kinds of effect exhibited different expression level, genetic diversity, expression variation, and expression diversity. For example, genes with both environment and haplotype effects had the highest genetic diversity, while genes with both haplotype and haplotype by environment effects had the lowest genetic diversity.

### The implication of population expression level and expression diversity under different environments

Since *E*_p_ and *E*_d_ were two indexes potentially weighting the roles in adaptation, we divided genes into 9 groups based on the changes of *E*_p_ and *E*_d_ (Supplementary Fig. S2). About 43% of transcripts were conserved in both *E*_p_ and *E*_d_. This group contained the genes that were insensitive to environmental change and were probably essential to plant growth and development. About 9% of transcripts had conserved *E*_d_ and upregulated *E*_p_, implying these genes had a common response to environment but without individual differentiation. About 24% of transcripts had upregulated *E*_p_ and enlarged *E*_d_. This group of genes probably had key functions for plant development and was easy to be artificially captured for adaptation (Supplementary Fig. S3).

## Discussion

### Expression level, genetic diversity and evolutionary rate

It is widely accepted that highly expressed genes evolve slowly^50^. Our result found that the genes without SNP tended to have higher expression level (Fig. 3a,b), which was consistent with previous conclusion. This could be explained by the stronger purifying selection for the highly expressed genes than those expressed at a lower level^51^. However, when we extended to study the relationship between expression level and genetic diversity, no correlation was observed when genetic diversity was at a low or middle level. Although we found a significantly difference of *E*_p_s for the last group having a high π value compared with the remained groups, this phenomenon may be caused by detection sensitivity for the genes with extreme high *E*_p_s in the last group. Thus the observed result that the genes with high genetic diversity had high *E*_p_s may be an illusion. Overall, our data suggested that the genetic contribution to differential expression level was mainly due to the presence or absence of SNP rather than the quantity of SNP.

Although there was very weak relationship between expression level and the quantity of SNPs in our data, we dissected the genetic variation of genes into nonsynonymous and synonymous variation. It was found a trend that higher proportion of nonsynonymous substitution was occurred in highly expressed genes (Supplementary Fig. S4). This phenomenon may be explained by that synonymous substitutions affect the stability of mRNA secondary structures and the genes with high mRNA level tend to need more stable secondary structures to prevent aggregation of mRNA molecules and improve the efficiency of translation elongation^52^,^53^. Thus, synonymous substitution in highly expressed genes may experience purifying selection. These suggested that strength of selection was different across expression level and the selection on the highly expressed gene was always stronger than the genes with low expression level.

### Negative relationship between expression diversity and genetic diversity

In our data, we found evidence for the negative relationship between expression diversity and genetic diversity, and even our newly sequencing data also support this conclusion. This seems inconsistent with some previous studies^54^. There was one explanation for the negative relationship between expression diversity and genetic diversity. *M. lutarioriparius* experienced chromosomal duplication in its evolution history^55^,^56^, therefore many genes had multiply copy number in genome. Duplicated genes often significantly increase gene expression diversity within and between species compared with single-copy genes^57^,^58^. However, duplicated genes have relatively weak genetic diversity compared with single-copy genes, since they evolve slower than single-copy genes^59^. Thus, the negative relationship between genetic diversity and expression diversity was observed in our results.

We speculated expression diversity may be an important contributor to adaptation to changing environment through the mechanism underlying phenotypic robustness, in which the process of development is balanced in an unpredictable world. Especially some potential adaptation-related genes with both low genetic diversity and high expression diversity had been detected in our previous studies^47^,^48^. Previous study found that reduced expression diversity within regulatory networks could promote the accumulation of genetic variation^60^. Thus we speculated that expression diversity had played the potential role in canalization to the environment. Under the balance of genetic variation and expression variation, *M. lutarioriparius* had experienced long-term natural selection and stayed at the state of canalization in its native region. In such case, this relationship between genetic and expression diversity could explain the genetic canalization mechanism that species had kept relatively invariant when individuals of the same single or multilocus genotype differ in their genetic background. Genes with higher expression diversity may have buffered the effects of lower genetic variation, and thus maintained the stability of phenotype in population consequently.

### Relationship between expression variation and genetic diversity

In our data, large expression variation (*E*_p_ ratio <  0.5 or *E*_p_ ratio >  2) was observed mainly in the genes without SNP, and *E*_p_ ratio tended to decrease with the increase of genetic diversity π (Fig. 5). This implied that the genes without SNP or having low genetic diversity were more susceptible to environmental changes in expression and were subjected to high environment effect^4^, which also supported that stress or environment responsive genes were often genetically conserved among populations and species^4^,^61^. Since the genes with low genetic diversity had high environment effect on expression, it can be inferred that gene expression variation was regulated by environment via trans-regulation.

In addition, out of the genes with and without SNP, 1.6% and 4.3% had *E*_p_ ratio of over 10, respectively, the extent to which may be considered as stress or environment responsive genes. Thus, these genes with large *E*_p_ ratio may represent adaptive responses to environmental change, and they were expected to experience purifying or strong positive selection and to harbor lower genetic diversity in population.

### Environment was the leading contributor to expression variation

Previous studies showed that environment was one of the most decisive factors on expression variation^4^. It was also argued that the environmental effect was stronger than the genetic effect^25^,^62^. To analyze the relative contribution of these factors, we utilized all transcript data and phased haplotype data respectively. We found the genes with environment effect were more frequent compared with those with population effect or haplotype effect, which supported that environment was the dominant contributor to gene expression variation. In our data, genetic diversity limitedly contributed to expression variation, and we found population stratification even had a much weaker role by comparing the relative proportion of genes with haplotype effect and population effect. This was consistent with the results of STRUCTURE analysis^48^, and suggested that although these plants had experienced long-period local adaptation in their native habitats, there was weak population structure and there had been frequent gene flow in these *M. lutarioriparius* populations^63^.

In our data, we found that environment was the leading contributor to expression variation compared with genetic diversity and population effects. It seems there was an amplification of environment effect and shrinkage of genetic effect and population effect compared with previous studies^64^,^65^,^66^,^67^,^68^. The main reason may be that the natural field conditions we adopted included much more variety of stresses than strictly controlled environment previous studies usually used. Since the haplotype had limited contribution to gene expression variation, we inferred that the adaptability to new environment was mainly attributed to some other factors rather than genetically induced adaptive genes, which was consistent with our result that environmentally induced expression diversity played a potential role in adaptation^48^.

### The implication of gene expression regulation in the process of adaptation to a new environment

Our previous study reported that gene expression diversity played a potentially positive role in adaptation to new environment by comparing the genetic diversity and expression diversity. We ranked Pfam families by the proportion of genes with enlarged *E*_d_ in QG, and found that the top five Pfam families were Legume lectin domain, WRKY DNA domain, 2OG-Fe(II) oxygenase superfamily, non-haem dioxygenase in morphine synthesis N-terminal, and Sugar (and other) transporter (Supplementary Fig. S3). The increased expression diversity of WRKY gene family, 2OG-Fe(II) oxygenase superfamily, and Sugar transporters, which could play important roles correlating with the environment, could help species to adapt to the changing environment^48^. Here we found high proportion of genes with environment and environment by haplotype (or genetic) effects on gene expression, which were stress responsive genes and local adaptation genes, respectively. Our data implied that gene expression mainly regulated by environment and haplotype contributed to adaptation to new environment. Our result may facilitate understanding of global gene expression variation in the process of plant adaptation to new environment and supply evidence for the contribution of gene expression variation to adaptation.

## Materials and Methods

### Data resource

The data were generated from our previous publication^48^. In brief, we collected *M. lutarioriparius* across its natural distribution and planted in two experimental fields, one near its native habitat JH and the other at the domestication site QG. The location of these materials had been described in previous study^48^. The same 14 populations of *M. lutarioriparius*, 3 individuals for each population were both randomly sampled in JH and QG. The fourth mature leaf of each individual at the same growth stage between two sites was chosen for RNA-seq. The 100-bp paired-end library was constructed and sequencing was performed on Illumina HiSeq 2000. Since in both sites 2 individuals were discarded due to the quality of their reads, a total of 40 individuals in each site were used for transcriptomic analysis ultimately. The raw data had been released at NCBI's Short Read Archive under three BioProjects, PRJNA227191, PRJNA227195, and PRJNA226258. We trimmed and filtered out reads showing more than 3% of bases with quality less than Q20. Then a high quality reference transcriptome was obtained using the population-based assembling method^48^. RNA-seq of *M. lutarioriparius* from each of the two experimental fields generated a total of ~2.76 billion 80 bp paired-end reads after the quality control. Sequence coverage for individual samples of the 80 individuals of *M. lutarioriparius* related to reference transcriptome ranged from 41.2% to 74.7%, with an average of 60.4% for the 80 individuals of *M. lutarioriparius*. Furthermore, the sequencing depth was saturated when the number of 80-bp reads of an individual used for assembly reached about 40 million^48^.

### Population expression level analysis

Clean reads of each individual were mapped to the Bowtie-build indexed reference transcriptome of *M. lutarioriparius* using TopHat and Cufflinks^69^,^70^. After excluding the genes with half individual with expression level equal to zero, 15367 genes remained. Expression level and population expression diversity were estimated as the formula 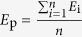 and 
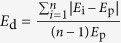
, where *n* represents the number of individuals, *E*_i_ represents the FPKM of a given gene of the *i*th individual in the population and *E*_p_ represents the expression level of a given gene. The expression level in population for genes in near native site JH and transplanted or target domestic site QG were abbreviated as *E*_p(JH)_ and *E*_p(QG)_ respectively, and the expression diversity for those genes were abbreviated as *E*_d(JH)_ and *E*_d(QG)_ respectively. The Wilcoxon test implemented in R 3.2.1 was applied to compare the gene expression level *E*_p_, gene expression diversity *E*_d_, and the ratio of *E*_p(QG/JH)_ among divided groups.

SNPs were identified using SAMtools with default settings^71^. After excluded SNPs with quality score ≤10, missing data ≥ 10% or minor allele frequency ≤ 0.05, the remained SNPs were used for further study. Genetic diversity (π ) for each gene of *M. lutarioriparius* was calculated based on remained SNPs. The genetic diversity for genes in JH and QG were abbreviated as π _(JH)_ and π _(QG)_ respectively. As the native site, JH was used as the denominator for calculating related ratios between two environments.

To distinguish the extent of conservation, we considered genes with *E*_p_ ratio ranged from 0.5 to 2 as conserved *E*_p_ genes, and genes with *E*_d_ change (*E*_d(QG)_–*E*_d(JH)_) of – 0.25 to 0.25 as conserved *E*_d_ genes.

### Haplotype inference and ANOVA analysis of haplotypes, population and environment effects

Population transcriptome SNP data sets were used to infer gene haplotype for individual from population data. We used PHASE v2.1.1to analyze the transcriptome data of the 80 individuals and to infer haplotypes^72^. PHASE was based on the approximate coalescent prior, which considered that the gene sequence of a mutant offspring differs only slightly from the progenitor sequence, and takes both homozygous and heterozygous positions into account when considering whether close-matching haplotypes are sought. Considering the high heterozygosity of *M. lutarioriparius* genome, we inferred the haplotype only for genes with less than 10 SNPs to ensure the accuracy of inference. For each gene, SNPs of all 80 individuals were pooled as input data for PHASE, and SNPs were ordered according to their relative position on the genes. The new model that makes explicit allowance for recombination was chosen for haplotype reconstruction with the default value for the initial estimate of the background recombination parameter of 0.0004. The program was implemented with default parameters (the default number of iterations is 100, with 100 burn-in, and a thinning interval of 1) to obtain the haplotype group with the highest probability. The haplotype group of best reconstruction with the highest probability in each gene was considered as the “best guess” estimate of the true underlying patterns of haplotype structure and was used in the analyses of haplotype expression.

Two-way ANOVA was carried out to study the environment effect and population effect on expression variation. In this analysis, the two-factor referred to the 14 populations and two environments. Additionally, since the specific haplotype of individuals for genes was inferred from population, a two-way ANOVA was also carried out to study the environment effect and haplotype effect on expression variation. Thus, in each gene, we fit a fixed effect general linear model including a term for population, haplotype, environment and their interaction. In all analyses, we controlled for multiple testing using a positive false discovery rate of 0.05.

## Additional Information

**How to cite this article**: Xu, Q. *et al.* Population transcriptomics uncovers the regulation of gene expression variation in adaptation to changing environment. *Sci. Rep.* **6**, 25536; doi: 10.1038/srep25536 (2016).

## Supplementary Material

Supplementary Information
