# Supplementary material for: Population transcriptomics uncovers the regulation of gene expression variation in adaptation to changing environment
Source: Sci Rep. 2016 May 6;6:25536. doi: 10.1038/srep25536 (PMC4858677; doi:10.1038/srep25536)
Supplement: Supplementary Information [file srep25536-s1.pdf]

Supplementary information

Population transcriptomics uncovers the regulation of gene expression variation in adaptation to changing environment

Qin Xu[1 §], Caiyun Zhu[2,4 §], Yangyang Fan[1,4], Zhihong Song[1,4], Shilai Xing[2,4], Wei Liu[2], Juan Yan[3] and Tao Sang[1,2*]

Supplementary Fig. S1 Comparison of E_p, E_d, π, E_p ratio, E_d ratio and F_{ST} among 8 types of effective genes. All genes were classified into 8 groups based on the genetic and/or environment effect in gene expression. G and E indicate the effect of haplotype and environment, respectively.

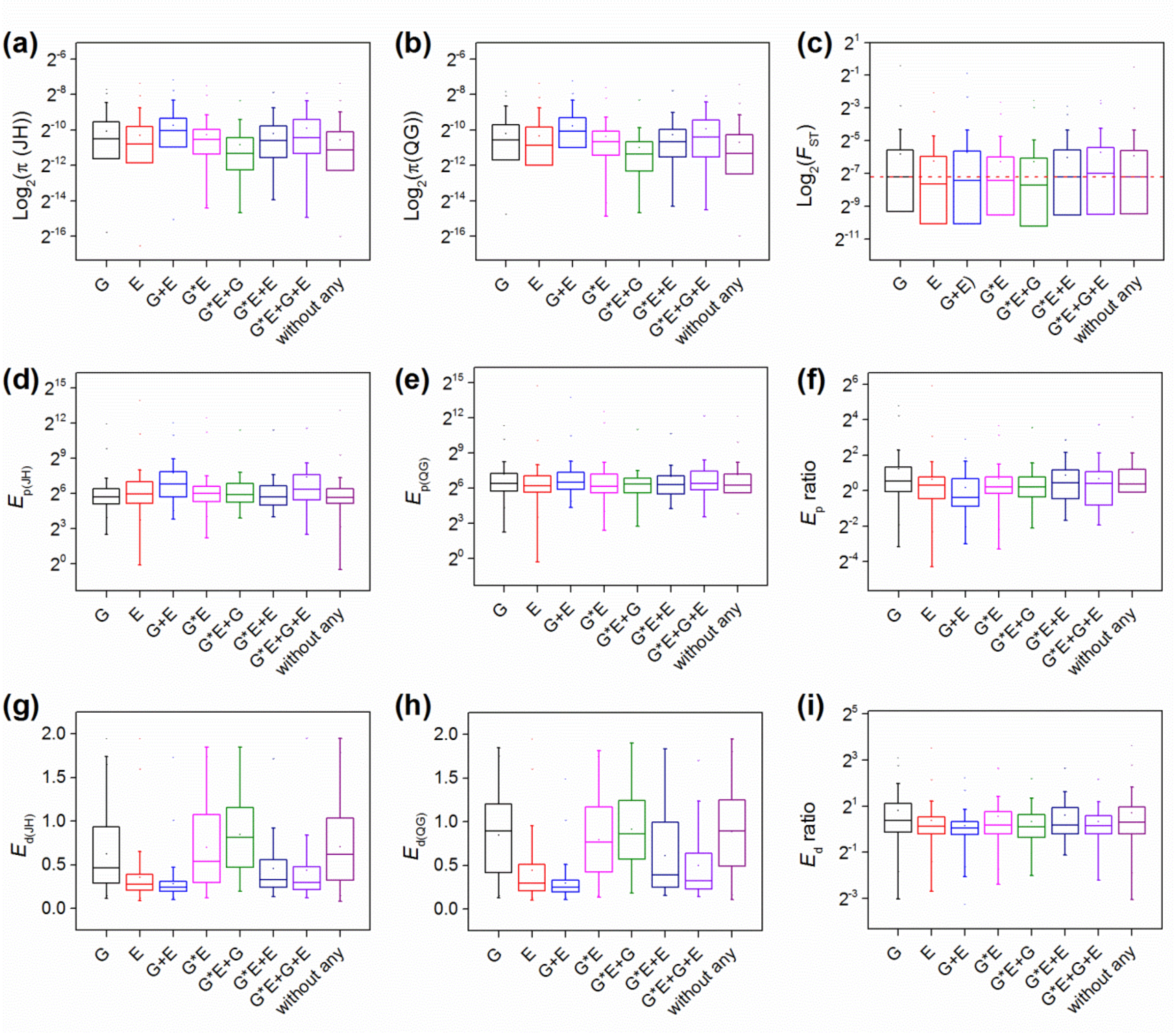

Supplementary Fig. S2 The distribution of E_p ratio and E_d difference between two environments. (a) Both of E_ps and E_ds were classified into three kinds of variation pattern, including up-regulated, down-regulated or conserved. The E_p ratio ranged from 0.5-2 was considered as conserved E_p, while E_p ratio larger than 2 and less than 0.5 were regarded as up-regulated and down-regulated separately. E_d difference less than 0.25 was considered as conserved E_d, while E_d difference larger than 0.25 and less than 0.25 were regarded as enlarge and shrink separately. Total transcripts were divided into 9 parts with the combinations of variation patterns of E_ds and E_ps. (b) Pie chart of proportion in 9 different expression variation patterns.

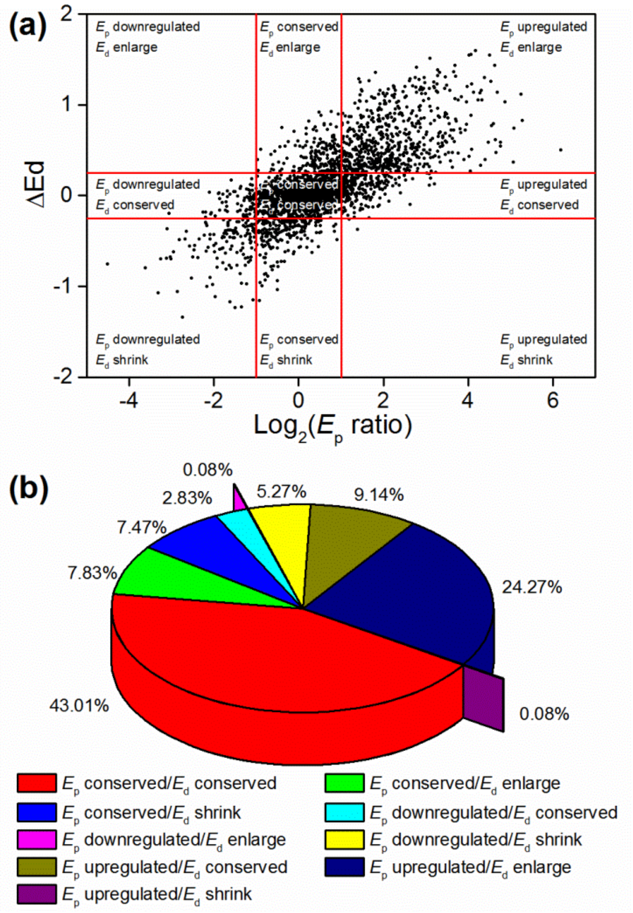

Supplementary Fig. S3 Function annotation of transcripts in 9 different expression patterns based on Pfam. Only pfam families with more than 40 members were considered. The relative proportion of transcripts in each pattern was shown in different colors.

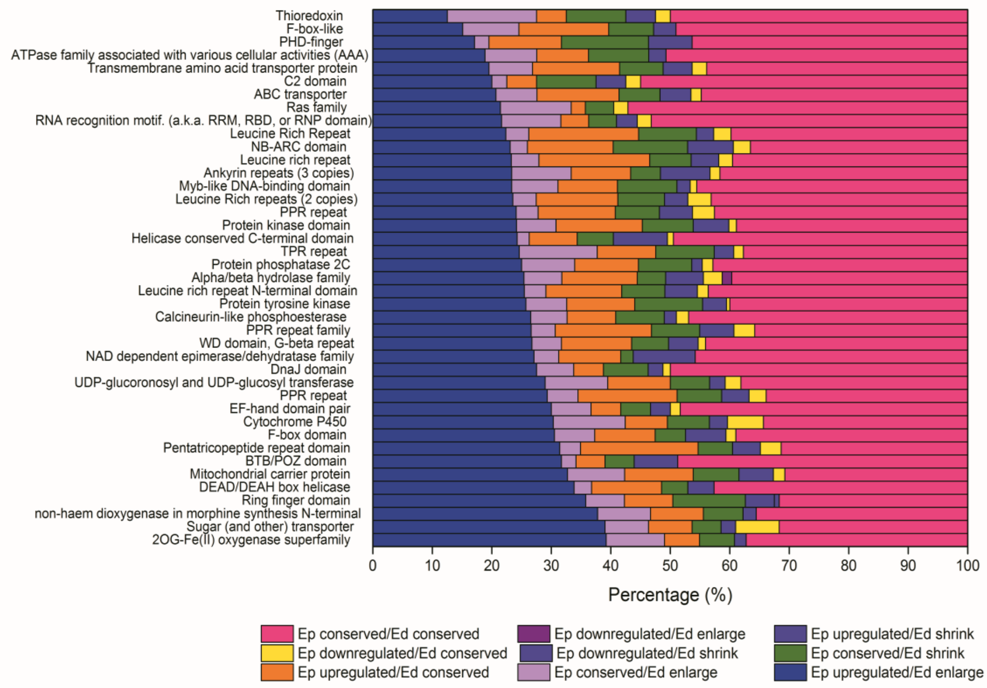

Supplementary Fig. S4 The proportion of two types of substitution under different $E_{ps.}$ Genes with SNP were selected and claasified into 8 groups based on the E_p in JH. SNPs in each group were calculated the proportion of nonsynonymous and synonymous substitution.

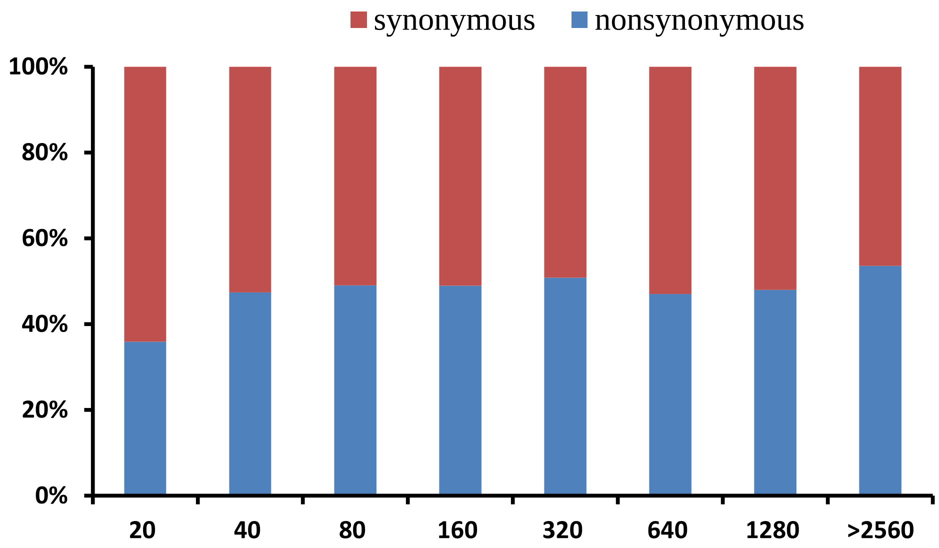

Supplementary Table S1 Pfam categories with high proportion of conserved E_ps

Pfam	Nonconserved	Conserved	Ratio
Ubiquitin-conjugating enzyme	5	30	0.86
FKBP-type peptidyl-prolyl cis-trans isomerase	3	17	0.85
Zinc finger, C3HC4 type (RING finger)	7	25	0.78
RNA recognition motif. (a.k.a. RRM, RBD, or RNP domain)	5	17	0.77
Ubiquitin family	6	19	0.76
Thioredoxin	10	30	0.75
SNF2 family N-terminal domain	7	20	0.74
Ras family	11	31	0.74
IQ calmodulin-binding motif	6	15	0.71
TCP-1/cpn60 chaperonin family	8	19	0.70
Triose-phosphate Transporter family	8	19	0.70
No apical meristem (NAM) protein	9	21	0.70
Pyridine nucleotide-disulphide oxidoreductase	6	14	0.70
Glycosyl hydrolase family 1	6	14	0.70
SWIM zinc finger	6	14	0.70
MYB-CC type transfactor, LHEQLE motif	6	14	0.70
EamA-like transporter family	10	23	0.70
ATPase family associated with various cellular activities (AAA)	21	48	0.70
bZIP transcription factor	12	26	0.68
Integrase core domain	8	17	0.68
RNA recognition motif. (a.k.a. RRM, RBD, or RNP domain)	55	116	0.68
MULE transposase domain	10	21	0.68
C2 domain	13	27	0.68
Alcohol dehydrogenase GroES-like domain	7	14	0.67
F-box-like	18	35	0.66
AMP-binding enzyme	12	23	0.66

RNA recognition motif (a.k.a. RRM, RBD, or RNP domain)	12	23	0.66
50S ribosome-binding GTPase	10	19	0.66
GRAS family transcription factor	10	19	0.66
CBS domain	9	17	0.65
Cyclophilin type peptidyl-prolyl cis-trans isomerase/CLD	8	15	0.65
Acetyltransferase (GNAT) family	8	15	0.65
Glycosyl transferases group 1	11	20	0.65
GDSL-like Lipase/Acylhydrolase	11	20	0.65
Glutathione S-transferase, N-terminal domain	10	18	0.64
DnaJ domain	29	51	0.64
PHD-finger	15	26	0.63
Myb-like DNA-binding domain	33	57	0.63
Major Facilitator Superfamily	14	24	0.63
PA domain	11	18	0.62
Kinesin motor domain	8	13	0.62
PB1 domain	8	13	0.62
DHHC palmitoyltransferase	8	13	0.62
Zinc-binding dehydrogenase	10	16	0.62
Calcineurin-like phosphoesterase	19	30	0.61
Protein phosphatase 2C	22	34	0.61
TPR repeat	24	37	0.61
EF-hand domain pair	24	36	0.60
Zinc finger C-x8-C-x5-C-x3-H type (and similar)	12	18	0.60
MatE	12	18	0.60
Total gene	6401	8961	0.58

Supplementary Table S2 Pfam categories of ANOVA results analysis of population effect, environment effect and population × environment interaction for 15367 genes.

Pfam	Annotation
PF00005[#]	ABC transporter
PF00067[¶]	Cytochrome P450
PF00069[#¶]	Protein kinase domain
PF00070[§]	Pyridine nucleotide-disulphide oxidoreductase
PF00076[#¶]	RNA recognition motif. (a.k.a. RRM, RBD, or RNP domain)
PF00201[¶]	UDP-glucoronosyl and UDP-glucosyl transferase
PF00226[¶]	DnaJ domain
PF00249[#¶]	Myb-like DNA-binding domain
PF00271[#¶]	Helicase conserved C-terminal domain
PF00400[#¶]	WD domain, G-beta repeat
PF00403[§]	Heavy-metal-associated domain
PF00560[#¶]	Leucine Rich Repeat
PF00931[#]	NB-ARC domain
PF00954[§]	S-locus glycoprotein family
PF01535[#¶]	PPR repeat
PF01545[§]	Cation efflux family
PF07714[#¶]	Protein tyrosine kinase
PF07716[§]	Basic region leucine zipper
PF13086[§]	AAA domain
PF13639[¶]	Ring finger domain

[#], [¶], and [§] indicates the variation caused by population effect, environment effect and population by environment interaction, respectively.

Supplementary Table S3 Summary of statistical results for genes with environment and haplotype by environment effect based on haplotypes of 6964 genes.

Genes Effective Type	Gene number (P<0.05)	Gene number (P<0.01)	π*1000 (QG)	π*1000 (JH)	E_p(JH)	E_p(QG)	E_p(QG)/E_p(JH)	E_d(JH)	E_d(QG)	E_d(QG)/E_d(JH)
G	629	504	0.65	0.69	51	84	1.65	0.46	0.89	1.93
E	1077	884	0.53	0.57	61	74	1.22	0.28	0.29	1.06
G * E	146	105	0.62	0.68	63	71	1.14	0.54	0.77	1.42
E + G	575	376	0.93	0.95	111	91	0.82	0.24	0.25	1.02
G*E + G	86	44	0.38	0.39	59	80	1.36	0.81	0.86	1.06
G*E + E	93	30	0.61	0.65	52	79	1.51	0.33	0.39	1.18
G*E + G +E	95	18	0.73	0.73	82	83	1.01	0.29	0.32	1.09

E: those exhibiting common expression environment responses; G: those exhibiting different expression among haplotype genotype in stress responses; G*E: those exhibiting haplotype-by-environment interaction effects on expression
